# Nurses’ and older patients’ perspectives on missed nursing care contextualised within the Fundamentals of Care Framework: A cross-sectional survey

**DOI:** 10.1016/j.ijnsa.2025.100452

**Published:** 2025-11-11

**Authors:** Anna Connolly, Anne Matthews, Marcia Kirwan

**Affiliations:** School of Nursing, Psychotherapy and Community Health, Dublin City University, Dublin, Ireland

**Keywords:** Fundamentals of care, Healthcare quality, MISSCARE Survey, Missed nursing care, Nursing, Older patients, Patient-reported outcomes, Patient safety

## Abstract

**Background:**

The Fundamentals of Care Framework outlines the core dimensions involved in delivering essential nursing care. Resource shortages and increased care demands compromise fundamental care delivery and contribute to missed nursing care. This impacts quality and safety within healthcare settings but is disproportionately experienced by older patients, therefore both nurse and patient voices must be heard.

**Objectives:**

To individually explore both nurse-reported and patient-reported perceptions of the frequency of missed nursing care. This research also aimed to estimate the factors that contribute to missed nursing care from nurses’ perspectives and to identify to what extent the *MISSCARE* instruments can represent the elements within the Fundamentals of Care framework.

**Design:**

A cross-sectional study using the *MISSCARE* instruments to elicit nurse and patient perspectives of missed nursing care.

**Setting:**

A single large university, tertiary hospital in Ireland with over 800 beds.

**Participants:**

Approximately 929 fully qualified nurses working in direct patient care and all patients aged 65 or older in 31 adult inpatient wards were invited to participate.

**Methods:**

The *MISSCARE Survey* and *MISSCARE Survey-Patient* were used to collect data between April and July 2024. Nurses indicated the frequency of and contributing factors to missed nursing care. Communication, timeliness and basic nursing care delivery were measured from the patients’ perspectives. The data were analysed using SPSS and mean scores were found for each care item. The items in the *MISSCARE* surveys were mapped to the elements in the Fundamentals of Care Framework.

**Results:**

A total of 151 patients and 145 nurses participated. According to nurses, attending interdisciplinary care conferences, mobilisation and oral care were frequently missed. Patients reported that oral care, communication in relation to who their specific nurse was and mobilisation were frequently missed. The significant reasons for missed care included inadequate numbers of nursing staff and assistive personnel and urgent patient situations. The *MISSCARE Survey-Patient* demonstrated a higher percentage coverage (73.7 %) of the elements outlined within the Fundamentals of Care framework than the *MISSCARE Survey* (42.1 %).

**Conclusions:**

This study reiterates the need to prioritise nurse recruitment and retention strategies and highlights areas which require attention to ensure the delivery of fundamental care. *The MISSCARE* surveys can measure the Fundamentals of Care Framework to a certain extent however, the development of a tool to directly measure all three framework dimensions is required. The development of a succinct tool to measure nurses’ and patient’s perspectives on missed nursing care is also required.

**Contribution of the paper**

What is already known about the topic

• The *MISSCARE Survey* and *MISSCARE Survey-Patient* are validated instruments that are used to elicit nurse and patient-reported data on the frequency of and reasons for missed nursing care.

• Older patients are particularly vulnerable to and disproportionately affected by missed nursing care due to their complex care needs resulting from co-morbidities and emergency admissions.

• Nurse shortages, a lack of resources and increased care demands contribute to missed nursing care and may result in adverse outcome for patients.

What this paper adds

• Including both patient and nurse perspectives of missed nursing care is important in order to promote person-centred care.

• Inadequate staffing is a significant contributor to missed nursing care, therefore nurse recruitment and retention strategies must be prioritised for safe and high-quality fundamental care to be delivered to older patients.

• The *MISSCARE* surveys can measure the Fundamentals of Care Framework to a certain extent however, further research is warranted to develop a tool to directly measure all three framework dimensions.

Alt-text: Unlabelled box

## 1 Background

Nurses play an integral role in the healthcare system through the organisation and delivery of care and are pivotal in promoting health and preventing illness (World Health Organization, 2024a). Accounting for approximately 59 % of all health professionals (World Health Organization, 2024b), nurses make up the largest occupational group in the healthcare system (World Health Organization, 2020). However, the estimated 4.5 million nurse shortage by 2032 (Boniol et al., 2022) is a challenge for healthcare systems worldwide. Nurse shortages, a lack of material resources, increased care demands and communication issues pose a threat to patient safety, a health priority which nurses contribute to by providing direct patient care (Phillips et al., 2021). These factors, coupled with issues relating to how fundamental care is valued within healthcare systems, can cause essential nursing care to be missed (Feo et al., 2019). Missed care is associated with costly health outcomes, compromised dignity, unsatisfactory experiences for patients and families and overall challenges to patient safety and welfare (Feo and Kitson, 2016).

The Fundamentals of Care framework outlines the three core dimensions involved in delivering high-quality and safe fundamental nursing care (Kitson et al., 2013) and provides a theory that can explain, guide and predict person-centred fundamental care (Kitson, 2018). The dimensions include a trusting nurse-patient relationship, the integration of care which ensures that patients’ physical, psychosocial and relational care needs are met and finally, the context of care which comprises of contextual factors that act as system and policy level enablers (Kitson et al., 2013).

There are currently no standardised measurement tools for tracking fundamental care (Kitson, 2018). However, identifying measurement tools has been highlighted as a research priority for challenging the perception of fundamental care as “common sense” (Feo et al., 2019, p. 82) and embedding a perception where the complexity of delivering high-quality fundamental care is recognised. Monitoring a patient’s fundamental care requirements and identifying areas where their needs have not been met could act as an early warning system to trigger a follow-up on patients’ care plans to ensure that they receive adequate care. Palese et al., 2021 explored the extent to which validated missed nursing care measurement tools can detect the elements within the Integration of Care dimension of the Fundamentals of Care framework. They identified a synergy between the framework and missed nursing care research lines and highlighted the potential of using missed nursing care tools to gather Fundamentals of Care framework metrics.

### 1.1 Missed nursing care model

Missed nursing care or care left undone refers to the regular omission or delay of required nursing care (Kalisch et al., 2009). Missed nursing care is underpinned by frequently missed care tasks such as ambulation, feedings, patient education, discharge planning, emotional support, hygiene, intake and output documentation and patient monitoring (Kalisch, 2006). The Missed Nursing Care Model (Kalisch et al., 2009) demonstrates how the demand for patient care, labour resources, material resources and communication interact with nursing processes such as assessments, planning, interventions and evaluation and are filtered by nurse’s internal processes such as group norms, priority decision making, internal beliefs and values and habits. The model outlines how the relationship between each of these attribute categories contributes to missed nursing care and highlights the impact of missed care on patient outcomes.

Use of the *MISSCARE Survey* has identified that fundamental nursing care activities are frequently missed (Du et al., 2020; Nahasaram et al., 2021). Growing reports of poorly executed Fundamentals of Care tasks, such as nutrition, hygiene, toileting and mobilisation (Feo and Kitson, 2016) which mirror the most frequently missed tasks in missed nursing care studies (Bagnasco et al., 2020) demonstrate the interrelation between the Missed Nursing Care Model and the Fundamentals of Care framework.

Although the *MISSCARE Survey* has been used to measure the Fundamentals of Care framework (Palese et al., 2021), the patient-reported tool has not been investigated in this context. Therefore, this study aimed to identify if the *MISSCARE Survey* and *MISSCARE Survey-Patient* can represent the elements outlined in the Fundamentals of Care framework. Furthermore, previous research only sought to measure the Integration of Care dimension of the framework (Palese et al., 2021) whereas this study explored the application of the *MISSCARE* measurement tools to all three framework dimensions.

Understanding and identifying missed nursing care in acute hospital settings allows for more targeted interventions to improve nursing care and ensure that fundamental care needs are met (Gallione et al., 2024). Furthermore, considering both nurses’ and patients’ perspectives is pivotal to providing interventions that promote person-centred care and align with the principles of the Fundamentals of Care framework (Kitson et al., 2013; Kitson, 2018).

### 1.2 Evidence of missed nursing care from nurses’ and patients' perspectives

Missed nursing care is commonly explored from the nurse perspective (Chiappinotto et al., 2022) with previous studies reporting care left undone on 45.3 % of shifts in adult acute wards and 46 % of shifts in older people’s wards (Senek et al., 2020). Furthermore, the prevalence of missed care across all nursing care elements ranges from 6.8 % to 98.1 % (Gong et al., 2025). Previous applications of the *MISSCARE Survey* (Kalisch and Williams, 2009; Dabney et al., 2019) identified turning, bathing, ambulation, emotional support and mouth care as frequently missed care activities (Cho et al., 2020). Additionally, mobilisation, assessing the effectiveness of medication (Gurková et al., 2024), attending interdisciplinary conferences and documentation are also frequently missed (Mainz et al., 2024).

Inadequate staffing, patient acuity, heavy admission and discharge activity, and urgent patient situations have previously been identified as significant reasons for missed nursing care (Mainz et al., 2024). Similarly, labour resources have been reported to significantly contribute to missed nursing care (Moreno-Monsiváis et al., 2015; Zelenikova et al., 2019). This demonstrates the association between inadequate labour resources, high demand for complex care and increased levels of missed nursing care (Cartaxo et al., 2024) and therefore highlights nurse staffing interventions as a priority for reducing missed nursing care.

Older patients, aged 65 and older, are vulnerable to missed care given their higher prevalence of co-morbidities and requirement of more complex nursing care (Merten et al., 2013). Older patients have a higher risk of developing complications and are more vulnerable to the consequences of care rationing (Bail and Grealish, 2016). Furthermore, frailty, which is common in older patients, with prevalence rates ranging from 48.8 % to 80 % (Rezaei-Shahsavarloo et al., 2020) is associated with missed care (Rezaei-Shahsavarloo et al., 2021).

Previous research using the *MISSCARE Survey-Patient* by Kalisch et al. (2014) found that patients reported more missed nursing care in the basic care domain than in the communication or timeliness domains. Frequently reported missed care items include mouth care, ambulation, assistance getting out of bed and into a chair, provision of information about tests and procedures and bathing. Ambulation is the most frequently omitted basic care task with 18.8 % of patients reporting that they never received help with ambulation (Gurková et al., 2024). Additionally, Sarpong et al. (2025) identified most missed care in the basic care domain, and highlighted the consideration of patients’ opinions as frequently missed. The communication domain has also received high mean subscale score with information regarding the assigned nurse reported as frequently missed (Cohen et al., 2024; Gurková et al., 2024). Furthermore, the most frequently patient-reported adverse events are IV-related problems, skin breakdowns, hospital-acquired infections and falls (Kalisch et al., 2014; Gurková et al., 2024; Sarpong et al., 2025).

### 1.3 Aim of this study

This is the first study in Ireland to explore both patients’ and nurses’ perceptions of missed nursing care, to our knowledge. Previous literature has focused on nurse perceptions of missed care with only three other studies investigating both patient and nurse perspectives (Moreno-Monsiváis et al., 2015; Cohen et al., 2024; Gurková et al., 2024). The importance of considering the patient’s perspective is evident (Bagnasco et al., 2020; Chiappinotto et al., 2022; Chiappinotto et al., 2023), therefore this study aims to individually explore and provide a narrative description of both nurse-reported and patient-reported perceptions of the frequency of missed nursing care, and estimate the factors that contribute to missed nursing care from the nurse perspective in an Irish hospital setting.

## 2 Methods

### 2.1 Design, setting and participants

This quantitative research utilised a cross-sectional study design to elicit both patient and nurse perspectives of missed nursing care in an acute hospital setting. The study was conducted in a single large university, tertiary hospital in Ireland with over 800 beds.

All fully qualified nurses employed across 31 adult inpatient surgical, medical and mixed surgical-medical wards were invited to participate. Student nurses, healthcare assistants and nurses employed in outpatient settings or nurses without direct patient care were excluded.

Patients aged 65 or older admitted into the same wards from which the nurses were recruited were eligible for inclusion. In order to capture an informed evaluation of care received, only patients with a minimum length of stay of three days were eligible for participation. Patients were required to be alert, oriented and cognitively capable of communicating, understanding and consenting to participate. This was established with the support of the nurse managers who identified eligible patients. Although the gatekeeping role of the nurse manager may have increased the risk of purposely excluding patients with negative perspectives, cognitive functioning can effect participants’ understanding of survey questions and instructions and their ability to provide accurate information (Kutschar et al., 2019) therefore, patients with declined cognitive functioning were not eligible for participation.

Nurses and patients were invited to participate via online and paper-based surveys between April 2024 and July 2024. Both the patient and nurse surveys could be accessed via QR codes which were printed on posters and information cards that were distributed across the hospital and within participating wards. Paper-based questionnaires, envelopes and sealed survey-return boxes were distributed to each participating ward.

### 2.2 Survey instruments

The *MISSCARE Survey* (Dabney et al., 2019) and *MISSCARE Survey-Patient* (Kalisch et al., 2014) were used to elicit nurse and patient perceptions of missed nursing care (supplementary material). The nurse questionnaire included a demographic information section followed by two sections exploring perceptions of missed nursing care. In Part A, nurses were asked to identify, using a five-point Likert scale (1-never missed, 2-rarely missed, 3-occasionally missed, 4-frequently missed, 5-always missed), how often 18 specific nursing care items were left undone on their unit. The *MISSCARE Survey* which was provided to the researchers by Dr. Beatrice Kalisch included a ‘not applicable’ option for Part A and was therefore retained as an option in the instrument used in this study. Part B consisted of 19 reasons for missed nursing care which were grouped into three subscales – communication, labour resources and material resources (Dabney et al., 2019; Zelenikova et al., 2019). Nurses were asked to assess the contribution of each reason to missed nursing care using a four-point Likert scale (1 = no contribution, 2 = minor contribution, 3 = moderate contribution, 4 = significant contribution). The survey instrument was also amended to include two additional questions relating to quality and safety. Nurses were asked to rate the overall quality of nursing care and grade the level of patient safety on their units.

The patient questionnaire included three sections. Firstly, patients were asked to identify to what extent various nursing care items were provided to them during their hospitalisation. A five-point Likert scale was used to measure items in the communication and basic care subscales (1 = never provided, 2 = rarely provided, 3 = sometimes provided, 4 = usually provided, and 5 = always provided). These questions were reverse coded, therefore higher scores indicated more missed nursing care. A five-point Likert scale was also used to measure items in the timeliness subscale (1 = <5 min, 2 = 5–10 min, 3 = 11–20 min, 4 = 21–30 min, 5 = >30 min) (Kalisch et al., 2014). The items were grouped into subscales (basic care, communication and timeliness) based on a 3-factor solution which emerged from an exploratory factor analysis conducted by Kalisch et al. (2014). Five additional questions relating to elements of nursing care were not included in these subscales and have been reported on separately. In the second section, patients were asked to identify whether they had experienced an adverse event, and the final section included demographic questions.

In addition to nurse and patients characteristics, the surveys addressed aspects of care such as eating and drinking, medication management, personal cleaning and dressing, toileting, safety, comfort and mobility. Nurses’ and patients’ overall rating of nursing care and perceptions of the frequency of missed nursing care were addressed via the surveys in addition to the reasons for missed nursing care according to nurses and patient-reported experiences of adverse events.

### 2.3 Data collection

Nurse recruitment was supported by the nursing executive, directorate nurse managers, clinical nurse managers and a clinical facilitator from the participating hospital. The support of the management in each ward facilitated the patient recruitment process. Permission was granted from the Director of Nursing to distribute recruitment posters and information cards to included wards, break rooms, changing rooms and other pertinent areas within the hospital. Permission was also granted for a researcher to be present on the wards to provide participation information. Furthermore, recruitment information was distributed via email to eligible nurses. Nurses had the option of accessing the survey online or completing a paper survey and returning it via the sealed survey-return boxes.

Permission was granted for patient recruitment posters and information cards to be distributed on the wards and throughout the hospital. Interested patients could access the survey via a QR code or could request a paper-based survey via the contact information on the posters. Paper-based surveys were distributed on the wards to interested patients. Additionally, permission was sought for the researcher to be present on the ward and to ask nurse managers for their support in identifying eligible patients and inviting them to participate. Similar to previous research by Kalisch et al. (2014) using the *MISSCARE Survey-Patient*, patients could fill out the survey themselves or if they had difficulty reading or writing, the researcher could read the questions to them and mark their answers.

### 2.4 Data analysis

After data cleaning, the data were analysed using IBM SPSS Statistics version 29. Descriptive statistics were used for data analysis. Frequencies were used to determine whether patients perceived each nursing care task as missed or not and the arithmetic mean, and standard deviation were used to summarise the data.

In part A of the *MISSCARE Survey*, nurses indicated the frequency of and contributing factors to missed nursing care. The mean missed nursing care score for each item and a mean overall score for missed care was calculated. In part B, an overall mean score for the factors that contribute to missed nursing care was calculated. Higher scores indicated more missed nursing care and more significant reasons for missed care. In line with (Kalisch et al., 2021), the non-applicable responses in part A were treated as missing data and were excluded with the mean scores for each missed care task calculated from the nurses who provided responses ranging from “never missed” to “always missed”. In order to analyse the frequency of missed nursing care and to report percentages of missed nursing care, the items in part A and part B were transformed into dichotomous variables as per the instructions for use of the survey (Kalisch et al., 2011; Kalisch et al., 2014). In line with previous applications of the *MISSCARE Survey* (Kalisch et al., 2011), items in part A were considered missed if they were reported as occasionally, frequently or always missed and not missed if they were reported as rarely or never missed. Similarly, and as demonstrated by Gallione et al. (2024), the items in part B were also considered dichotomously. Reasons that were reported to have a significant or moderate contribution to missed nursing care were considered significant and reasons reported to have a minor or no contribution were considered as non-significant reasons.

Communication, timeliness, and basic nursing care delivery were also measured from the patients' perspectives. An overall mean score for missed nursing care for each of these subscales according to the patient perspective was calculated, again with higher scores indicating more missed care. As demonstrated by Kalisch et al. (2014), the items in the *MISSCARE Survey-Patient* were also considered dichotomously in line with part A of the nurse questionnaire. The responses ‘never’, ‘rarely’ and ‘sometimes’ were considered as missed and the responses ‘usually’ and ‘always’ were considered not missed (Sarpong et al., 2025). Patients were also asked to report whether they had experienced specific adverse events using the responses ‘yes’, ‘no’ or ‘unsure’. In line with analysis methods used by Sarpong et al. (2025), the ‘unsure’ responses were excluded during data analysis.

Furthermore, the items measured in the MISSCARE surveys were mapped to the elements in the Fundamentals of Care Framework. In order to understand the extent to which the *MISSCARE* instruments can be applied to the Fundamentals of Care framework, the elements outlined within the framework and the surveys were considered and a comparative analysis was conducted. The elements in the framework were extracted and presented in a tabular format (Table 6). The items measured within the *MISSCARE Survey* and *MISSCARE Survey-Patient* were also extracted, analysed and presented within the table to demonstrate their correspondence to the elements in the framework. The elements within the framework were considered in line with the definitions provide by Kitson et al. (2013). This process was conducted by one researcher and reviewed by two senior members of the research team. Consensus was reached in relation to the correspondence of the items and in line with previous research from Palese et al. (2021), a scoring system was used to conduct a quantitative assessment of coverage to demonstrate the percentage of each of the dimensions of the Fundamentals of Care Framework which is covered by *the MISSCARE Survey* tool and the *MISSCARE Survey-Patient* tool. A score of 0 was given when there was no correspondence and a score of 1 was given for each survey item which corresponded with an element of the Fundamentals of care Framework.

### 2.5 Ethical considerations

Ethical approval was obtained from the Dublin City University Research Ethics Committee and the participating hospital’s Institutional Review Board. The study was conducted in accordance with the Declaration of Helsinki (World Medical Association, 2018). Approval was sought from the Director of Nursing and the nursing executive in the participating hospital to approach nurse managers on the wards and ask their permission to access both nurses and patients and invite them to participate in the surveys. All interested participants were provided with information regarding the study and informed that the surveys were voluntary. Informed consent was implied when the surveys were completed and returned. Nurse managers also granted permission at ward level for nurses to complete the questionnaire whilst on shift.

## 3 Results

### 3.1 Participants

A total of 151 nurses and 145 patients met the inclusion criteria, completed and submitted a questionnaire.

#### 3.1.1 Methodological considerations

Although it is unclear how many nurses were reached via the recruitment advertisements and invited to participate, there were approximately 929 nurses who were eligible to participate. The response rate was 16.3 % with 151 nurses completing and submitting the survey. It was not possible to calculate a response rate for the patient survey as the number of patients who met the inclusion criteria was unclear due to patient level variation across the study period. Also, as recruitment advertisements were placed around the hospital, it is unclear how many potential participants were reached. According to the healthcare pricing office, approximately 6500 patients were admitted to the participating hospital that year however the exact number of inpatients at the time of data collection is unknown. The maximum margin of error recommended in survey research is 10 %, and when considering a population of 1000 and 10,000, a minimum sample of 88 and 96, respectively, is required (Serdar et al., 2021), therefore a minimum of 100 patient participants was considered appropriate.

The characteristics of the nurse and patient participants are reported in supplementary Tables 1 and 2, respectively.

Furthermore, data pertaining to the number of nurses and healthcare assistants who provided care, the number of patients cared for, the number of admissions and discharges and the number of patients emergencies on the wards within which the nurses who participated in the survey worked are presented in supplementary Table 3.

### 3.2 Understanding nurse and patient-reported perceptions of missed nursing care

In order to understand both the nurse and patient-reported perceptions of the same items of missed nursing care, and to demonstrate their correspondence with the discrete elements of care outlined in the Fundamentals of Care framework, the results of the surveys are presented in Table 1 using the Fundamentals of Care framework headings.

**Table 1:** Nurse and patient perceptions of missed nursing care contextualised within the Fundamentals of Care Framework.

Fundamentals of Care Framework category	Nurse	Missed	n	Patient	Missed	n
Eating and drinking	Patients fed while food is still at the proper temperature*	34 %	144	Timely assistance with feeding **	0 %	22
	Setting up meals for patient who can feed themselves*	28.6 %	140			
Medication Management	Medications administered as scheduled and on time*	16.2 %	148	Correct medication given****	3.9 %	129
	PRN medications requests acted on within 15 min*	18.8 %	149	Medication given on time****	6.2 %	130
				Pain medication received promptly after request***	5.6 %	71
				Nursing staff checked back to see if the medication helped reduced pain**	36.5 %	74
Personal Cleansing and dressing	Bathing/showering*	24.8 %	149	Bathing/showering**	28.1 %	139
	Oral care*	41.5 %	147	Oral care**	64.4 %	135
Safety	IV/central line site care and assessments according to hospital policy*	15.6 %	147	IV or other line (central venous catheter, PICC line, or port) checked by nurses**	7.9 %	127
Toileting	Assist with toileting needs within 5 min of request*	30.4 %	148	Assisted with toileting within 5 min of request*****	22.2 %	54
Comfort	Response to call light is initiated within 5 min*	30.7 %	140	Response to call light within 5 min*****	13.6 %	110
				Help received within 5 min of call light being answered*****	14.8 %	108
Mobility	Mobilisation*	41.7 %	146	Nursing staff monitored/assisted with getting out of bed and into a chair**	26.9 %	130
				Nursing staff monitored/assisted with walking**	38.5 %	109

*The responses ‘occasionally missed’, ‘frequently missed’ or ‘always missed’ were considered missed and the responses ‘rarely missed’ or ‘never missed’ were considered not missed.

**The responses ‘never provided’, ‘rarely provided’ and ‘sometimes provided’ were considered as missed and the responses ‘usually provided’ and ‘always provided’ were considered as not missed.

***The responses ‘<5 min’, ‘5–10 min’ and ‘11–20 min’ were considered as care that was not missed and was delivered promptly whereas the responses ’21–30 min', ‘>30 min’ and ‘never received the pain medication’ were considered as missed.

****The response ‘yes’ was considered as not missed and the response ‘no’ was considered missed.

*****The response ‘<5 min’ was considered as care that was not missed and was delivered promptly whereas the responses ‘5–10 min’ and ‘11–20 min’, ’21–30 min' and ‘>30 min’ were considered as missed or delayed.

### 3.3 Overall rating of nursing care, staffing and safety

The overall rating of nursing care was considered dichotomously (poor-fair and good-excellent). The nurses were asked to rate the overall nursing care using a four-point Likert scale (1 = poor, 2 = fair, 3 = good, and 5 = excellent) and the patients were asked to rate their nursing care using a five-point Likert scale (1 = poor, 2 = fair, 3 = good, 4 = very good, and 5 = excellent). The majority of both nurses (85.9 %) and patients (97.9 %) reported that overall nursing care was good-excellent. A total of 13.4 % of nurses perceived nurse staffing adequacy 100 % of the time and 29.5 % of nurses rated the patient safety grading on their ward as excellent.

### 3.4 Nurse perceptions of the frequency of missed nursing care

The mean missed nursing care scores for each item is presented in Table 2. The overall mean score falls between ‘never missed’ and ‘rarely missed’, indicating that nurses reported a low incidence of missed nursing care. Attending interdisciplinary care conferences followed by mobilisation and oral care were most frequently missed.

**Table 2:** Mean scores indicating the frequency of missed nursing care according to nurses' perceptions.

Nurse perceptions of the frequency of missed nursing care		
Items	Mean	SD
Assessment		
Full documentation of all care provided	2.06	1.05
IV/central line site care and assessments according to hospital policy	1.65	0.87
Monitoring intake/output	1.89	0.93
Assessment of vital signs	1.43	0.68
Glucose monitoring as ordered	1.46	0.72
Adequate surveillance of cognitively impaired patients	1.97	0.98
Interventions – Individual Needs		
PRN medications requests acted on within 15 min	1.82	0.94
Medications administered as scheduled and on time	1.68	0.83
Assist with toileting needs within 5 min of request	2.07	1.06
Response to call light is initiated within 5 min	2.09	1.08
Interventions – Basic Care		
Mobilisation	2.47	2.47
Pressure relieving interventions	2.11	1.04
Oral care	2.29	1.09
Feeding patients while food is still at the proper temperature	2.17	1.04
Setting up meals for patient who can feed themselves	1.99	1.07
Bathing/showering	1.99	0.98
Skin/wound care	1.76	0.83
Planning		
Attend interdisciplinary care conferences when held	2.54	1.33
Mean score	1.97	.74

Note: A five-point Likert scale was used (1-never missed, 2-rarely missed, 3-occasionally missed, 4-frequently missed, 5-always missed).

### 3.5 Nurse perceptions of the factors that influence missed nursing care

The reasons for missed nursing care are presented in Table 3. Overall, labour resources contributed most significantly to missed nursing care. Inadequate numbers of nursing staff and assistive personnel were identified as significant contributors to missed nursing care. A total of 87.2 % and 83.6 % of nurses, respectively, identified these as significant reasons for missed nursing care. Furthermore, 87.2 % of nurses highlighted that urgent patient situations significantly contribute to missed care (Supplementary Figure 1).

**Table 3:** Mean score indicating the contribution of each reason to missed nursing care according to nurses' perceptions.

Reasons for missed care		
Items	Mean	SD
Communication		
Unbalanced patient assignments	2.91	0.97
Inadequate hand-over from previous shift or transferring ward/unit	2.73	0.95
Other departments did Not provide the care need (e.g., physiotherapy, occupational therapy, medical social work)	2.72	0.94
Lack of back up support from team members	2.71	1.01
Tension or communication breakdowns with other staff/departments	2.72	0.95
Tension or communication breakdowns within the nursing team or with the medical staff	2.77	0.98
Inadequate supervision of healthcare assistants	3	1.01
Mean score	2.80	0.78
Material Resources		
Medications Not available when needed	2.81	0.90
Supplies/equipment Not available when needed	2.74	0.92
Supplies/equipment Not functioning properly when needed	2.76	0.89
Mean score	2.77	0.81
Labour Resources		
Inadequate number of staff	3.47	0.79
Urgent patient situations (e.g., a patient's condition worsening, patient fall)	3.36	0.79
Unexpected rise in acuity on the ward/unit	3.30	0.81
Inadequate number of assistive personnel (e.g., healthcare assistants, etc.)	3.40	0.88
Heavy admission and discharge activity	3.25	0.86
Emotional or physical exhaustion	3.27	0.88
Interruptions/multitasking	3.29	0.91
Inadequate support from nursing leadership	2.67	1.07
Mean score	3.25	0.67

Note: A 4-point Likert scale was used. (1-no contribution, 2-minor contribution, 3-moderate contribution, 4-significant contribution).

### 3.6 Patient perceptions of missed nursing care in the context of communication, timeliness and basic care delivery

The patients’ perceptions of missed nursing care are presented in Table 4. The basic care subscale had the highest mean subscale score. The most frequently missed item in this domain was mouth care, followed by ambulation. The analysis of the individual items indicated that items within the communication domain were more frequently missed. Patient-reported experiences of adverse events are presented in Table 5.

**Table 4:** Means scores indicating patients perceptions of the frequency of missed nursing care.

Missed nursing care events from patients’ perspective		
Items	Mean	SD
Communication		
Clear about assignment of specific nurse	2.68	1.71
Nursing staff discussed treatment	2.07	1.32
Nursing staff provided information about tests/procedures	2.17	1.42
Questions or concerns about care or illness listened to by nursing staff	1.43	0.94
Opinions relative to care considered by nursing staff	1.81	1.16
Mean score	2.062	0.92
Timeliness		
Time taken for nursing staff to respond to monitor or other machine beeping	1.45	0.93
Time taken for nursing staff to respond to call light	1.2	0.59
Time taken for nursing staff to provide help in response to the call light	1.19	0.53
Time taken for nursing staff to provide help to go to the bathroom	1.26	0.52
Mean score	1.33	0.71
Basic Care		
Mouth care	3.44	1.73
Bathing/shower care	1.97	1.44
Assistance to get out of bed and sit in a chair	2.03	1.47
Ambulation	2.37	1.62
Mean score	2.43	1.23

Notes: A five-point Likert scale was used to measure items in the communication and basic care subscales (1 = Always, 2 = Usually, 3 = Sometimes, 4 = Rarely, and 5 = Never).

**Table 5:** Patient-reported experiences of adverse events and overall rating of nursing care.

Prevalence of adverse events			
Adverse events	**Yes *%***	**No *n (%)***	**Unsure *n (%)***
Fall	6.3 %	93 %	0.7 %1`
Pressure Ulcer	7.0 %	92.3 %	0.7 %
Medication error	3.6 %	89.2 %	7.2 %
Hospital-acquired infection	21 %	79 %	-
Problems with IV cannula	20.6 %	77.9 %	1.5 %

**Table 6:** Similarities between the items addressed within the missed nursing care model and the Fundamentals of Care Framework.

		MISSCARE Survey	MISSCARE Survey-Patient
Number of items/elements			
	**Context of Care**		
Policy Level	Financial issues	0	0
	Quality and Safety	3	8
	Governance issues	0	0
	Regulation and accreditation	0	0
	Percentage ( %) of the policy level of the Context of Care dimension covered by tools' items	25.0	25.0
System Level	Resources	6	0
	Evaluation and feedback	0	0
	Leadership	1	0
	Culture	5	0
	Percentage ( %) of the system level of the Context of Care dimension covered by tools' items	75.0	0.0
	**Integration of Care**		
Physical care recipient actions	Rest and sleep	0	0
	Personal cleansing and dressing	2	2
	Medication management	3	5
	Toileting needs	1	1
	Eating and drinking	2	1
	Comfort (comfort interventions such as pressure relieving, feeding, pain relief, hygiene, responses to patient requests)	10	12
	Safety	25	8
	Mobility	1	2
	Percentage ( %) of the physical aspects of the Integration of Care dimension covered by tools' items	87.5.0	87.5
Psychosocial care recipient actions	Communication	5	6
	Being involved and informed	0	6
	Respect	0	2
	Education and information	0	5
	Having beliefs and values considered and respected	0	2
	Dignity	3	5
	Emotional wellbeing	0	2
	Privacy	0	0
	Percentage ( %) of the psychosocial aspects of the Integration of Care dimension covered by tools' items	25.0	87.5
Relational caregiver actions	Being empathetic	0	2
	Helping care recipients to cope	0	7
	Engaging with care recipients	0	6
	Supporting and involving families and carers	0	0
	Working with care recipients to set goals	0	1
	Active listening	0	2
	Helping care recipients to stay calm	0	1
	Being compassionate	0	2
	Being present	0	6
	Percentage ( %) of the relational aspects of the Integration of Care dimension covered by tools' items	0.0	88.9
	**Relationship**		
	Trust (provision of information, responding to patient requests)	3	8
	Focus (surveillance anticipation, detecting changes)	5	11
	Anticipate	6	11
	Know	0	2
	Evaluate	0	5
	Percentage ( %) of the relationship dimension covered by tools' items	60.0	100.0
Total	Overall coverage percentage ( %) of the three dimensions covered by tools' items	**42.1**	**73.7**

### 3.7 Mapping of the MISSCARE surveys to the elements in the Fundamentals of Care Framework

Based on an evaluation of the correspondence between the elements within the Fundamentals of Care framework and the *MISSCARE* survey instruments using Kitson et al.’s (2013) framework definitions, this research suggests that the *MISSCARE Survey* can almost fully measure the system level elements within the Context of Care dimension and the physical care recipient actions within the Integration of Care dimension. The policy level elements within the Context of Care dimension, the psychosocial care recipient actions within the Integration of Care dimension and the Relationship dimension are partially measured whilst the relational caregiver actions within the Integration of Care dimension are not directly measured. Overall, the *MISSCARE Survey* tool can measure approximately 42 % of the elements outlined within the Fundamentals of Care Framework.

The ability of the *MISSCARE Survey-Patient* to measure the Fundamentals of Care Framework was also evaluated. The physical care recipient actions, psychosocial care recipient actions and the relational caregiver actions within the Integration of Care dimension and the Relationship dimension are almost fully measured. The policy level elements within the Context of Care dimension are partially measured and the system level elements are not directly measured. Overall, the *MISSCARE Survey-Patient* can measure over 73 % of the elements outlined within the Fundamentals of Care Framework.

## 4 Discussion

This is the first study in Ireland to measure missed nursing care from both patients’ and nurses’ perspective using the *MISSCARE* surveys. Including both nurses’ and patients’ perspectives ensures that nursing care is person-centred and focused on patients’ needs (Bagnasco et al., 2020). The results of this study suggest that nurses perceive a low incidence of missed nursing care with an overall low missed nursing care mean score. Similar to findings from Nahasaram et al. (2021), these results fall between the ‘never’ and ‘rarely missed’ categories. According to patients, basic care tasks were most frequently missed however, some items within the communication subscale received higher mean scores indicating that they are also frequently missed. The results demonstrate that, according to nurses, labour resources contribute significantly to missed nursing care. Additionally, the results demonstrate the extent to which the Fundamentals of Care Framework can be measured by the *MISSCARE* instruments.

### 4.1 Nurse perceptions of the frequency of missed nursing care

Previous research has also reported low mean scores of missed nursing care from the nurse perspective (Kalisch et al., 2013; Cho et al., 2015; Zelenikova et al., 2019; Gurková et al., 2024). Furthermore, missed nursing care research from Turkey, Australia, the United States and Iceland identified an overall low occurrence of missed nursing care with a mean score indicating that care is missed rarely-occasionally, although the highest mean score for missed nursing care was found in Turkey with care being missed occasionally-frequently (Bragadóttir et al., 2020). Although this study identified low rates of missed care, the association between labour resources and missed care demonstrates the importance of focusing on nurse retention strategies to ensure adequate staffing and prevent missed nursing care. The correlation between nurses’ intention to leave and missed nursing care further emphasises the need to promote nurse retention. Workload, burnout, managerial support and job satisfaction are key areas which require consideration when developing retention strategies. In order to reduce missed nursing care and improve the sustainability of the nursing profession, targeted interventions that improve nurses’ working conditions are required. Retention strategies that address staffing shortages, improve workloads and encourage managerial support are required (Azzellino et al., 2025).

### 4.2 Nurse perceptions of the factors that contribute to missed nursing care

The results of this study reflect previous reports of shortfalls in labour resources significantly contributing to missed nursing care according to nurses (Zelenikova et al., 2019; Cartaxo et al., 2024). Similarly, Mainz et al. (2024), Moreno-Monsiváis et al. (2015) and Chiappinotto et al. (2023) found that inadequate staffing was perceived by both nurses and patients to be the most significant reason for missed care. These results are consistent with previous literature relating to the global shortage of nurses within the healthcare workforce (World Health Organization, 2020). Lower nurse-to-patient ratios is associated with rationed nursing care and poor patient outcomes (Zhu et al., 2019). In particular, the impact of staff shortages on nurses’ ability to mobilise older hospitalised patients has been highlighted. This emphasises the need to recognise the harmful effects of poor nurse-to-patient ratios on mobilisation of older hospitalised patients (Lim et al., 2020) and reiterate the need to establish effective nurse workforce planning processes, both in Ireland and globally, that focus on retaining experienced nurses and ensuring that nursing is seen as a valued and attractive profession (Morris et al., 2019).

### 4.3 Patient perceptions of the frequency of missed nursing care

While nurses reported that care was never or rarely missed, patients reported that basic care and communication subscale items were only usually or sometimes delivered and highlighted frequently missed items such as oral care. This finding is in line with previous reports from both nurses’ and patients’ perspectives (Kalisch et al., 2014; Cho et al., 2020; Cohen et al., 2024; Gurková et al., 2024; Sarpong et al., 2025). Given the relationship between oral care and systemic conditions such as diabetes, cardiovascular disease and dementia (Kapila, 2021), and the generally poor oral health among older patients in Ireland (Sheehan et al., 2017) and internationally (Gil-Montoya et al., 2015), oral care must be prioritised in terms of a patient safety agenda. The adoption of oral care education programmes for both patients and nurses and the establishment of formal oral care protocols in addition to nurse managers support of implementing care standards are evidence-based solutions to improving oral care practices in hospital settings (Gallione et al., 2024).

Mobilisation was also identified as a most frequently missed care task. Nurses often perceive mobilisation to be the responsibility of the physiotherapist and can lack confidence in their mobilisation skills and knowledge. However, given their daily provision of direct patient care, their role in promoting mobilisation of older patients is important. Investment from healthcare organisations in mobilisation education and training may empower nurses to take responsibility for mobilisation of older patients and improve patient outcomes (Constantin and Dahlke, 2018). A nurse-driven mobility protocol which proved effective in increasing out-of-bed episodes, improving patient mobility and reducing length of stay has been developed by Li et al. (2025). Implementing a nurse-driven mobilisation protocol may offer an evidence-based solution to this issue and prevent the functional decline of older patients in acute hospitals. Additional evidence-based solutions to improve mobilisation and reduce missed care in general include ensuring adequate nurse-patient ratios, promoting adherence to guidelines and protocols, engaging nurses in missed nursing care issues by providing education and training and redesigning nursing documentation by developing time-efficient, smart electronic recording with reminder systems to prompt nurses to provide aspects of care such as mobilisation (Longhini et al., 2021).

Similar to previous research, vital signs assessments and glucose monitoring were identified as aspects of patient care that were least frequently missed (Kalisch et al., 2011). These elements of care may be missed less frequently than others given that it is more evident when they are not carried out. Routinely documented aspects of care may be less likely to be missed in comparison to tasks which are not routinely recorded in nursing documentation and therefore may be less likely to be noticed when missed. The routine auditing of aspects of care such as assessments of vital signs and glucose monitoring may also contribute to the lower frequency at which these items are left undone. Furthermore, these care tasks may be placed higher on the priority list and completed more promptly than care tasks such as ambulation, which are more time-consuming and may require additional members of staff (Kalisch et al., 2011). These findings are in line with research relating to the Fundamentals of Care in clinical practice. According to Pene et al. (2025) nurse-patient interactions are often focused on completing tasks within the physical aspects of the Fundamentals of Care framework, with tasks relating to the relational and psychosocial aspects of care missed more frequently.

### 4.4 Using the MISSCARE surveys to measure both patient and nurses perspectives of missed nursing care

Few previous studies have reported on both nurse and patient perceptions of missed nursing care. As mentioned, differences between the items measured in the *MISSCARE Survey* and *MISSCARE Survey-Patient* limits the ability to make direct comparisons between nurse and patient perceptions of missed nursing care (Cohen et al., 2024). However, the *MISSCARE Survey* and *MISSCARE Survey-Patient* can be used to measure nurse and patient perspectives of the same phenomena in terms of the frequency of certain items of missed care (Table 1). Eliciting patients’ and nurses’ perceptions has been identified as an important aspect of reporting missed nursing care (Bagnasco et al., 2020). Therefore, future research should aim to include both viewpoints to respond effectively to nurses’ and patients’ needs. Again, to allow for a more direct comparison between patient and nurse perceptions of missed nursing care, the development of a tool to measure both perspectives is warranted.

The lack of statistically significant correlations between nurse-reported and patient-reported overall missed nursing care scores (Cohen et al., 2024; Gurková et al., 2024) suggests that patients and nurses have differing perceptions of missed nursing care and emphasises the need to understand both perspectives (Bagnasco et al., 2020). This lack of correlation may be attributable to the use of different survey instruments, the *MISSCARE Survey* and the *MISSCARE Survey-Patient*, which may measure different phenomena. However, Moreno-Monsiváis et al. (2015) used the same *MISSCARE Survey* to measure missed nursing care from both patients’ and nurses’ perspectives and found no significant associations. This suggests that the lack of correlation is more likely to represent true differences between nurses’ and patients’ perspectives of missed nursing care.

There are several potential reasons for the differences between nurse and patient perspectives of missed nursing care. According to Gustaffson et al. (2020), differences may arise as patients may have difficulty distinguishing between nurses and other healthcare providers and their responsibilities. Also, patients’ and nurses’ recognition of care needs may not align and therefore may contribute to differences of opinions in relation to the extent to which care is provided. Furthermore, previous research has demonstrated that patients may not recognise all the care tasks for which nurses are responsible, given that they tend to recognise the more medical aspects of care such as medication surveillance (Kalisch et al., 2012). Differences pertaining to nurse and patient priorities may also contribute to the divergence between their perspectives of missed care. Patients appear to place higher value on the emotional and psychological aspects of care whereas nurses tend to place more emphasis on medical aspects of care (Bagnasco et al., 2020).

### 4.5 Mapping the items measured in the MISSCARE to the elements in the Fundamentals of Care Framework

The results of this study demonstrate that some of the most frequently missed nursing care activities are addressed in the Fundamentals of Care framework. This highlights the overlap between the Missed Nursing Care Model and the Fundamentals of Care framework and demonstrates the potential for the *MISSCARE* surveys to be used to measure certain elements of the Fundamentals of Care framework.

Similar to research conducted by Palese et al. (2021), this study has highlighted the similarities and divergencies between the elements of care addressed in the Fundamentals of Care framework and in the *MISSCARE* surveys. As previously identified, the *MISSCARE Survey* directly measures most elements of care within the physical of the integration of care dimension. However, in contrast to previous research, this study sought to explore the application of both the *MISSCARE Survey* and *MISSCARE Survey-Patient* to all three dimensions of the Fundamentals of Care framework. As evident from this research, the *MISSCARE Survey* and the *MISSCARE Survey-Patient* can be used to a certain extent to measure certain elements outlined within the Fundamentals of care Framework. The *MISSCARE Survey-Patient* can be used to elicit patient perspectives on the physical, psychosocial and relational care actions in addition to the relationship between the nurse and patient which is a crucial component in the delivery of fundamental nursing care (Kitson et al., 2013). This research demonstrates that the MISSCARE Survey-Patient provides a more comprehensive measurement of the Fundamentals of Care Framework than the *MISSCARE Survey* and therefore highlights the limitations of the use of the *MISSCARE Survey* to measure the delivery of fundamental care. Although this demonstrates some convergences between the Fundamentals of Care Framework and the Missed Nursing Care model, there are substantial coverage gaps, particularly within the *MISSCARE survey*, that highlight the demand for the development of a tool that can be used to directly measure all elements of the Fundamentals of Care Framework.

### 4.6 Strengths and limitations

The inclusion of only one hospital site is a limitation of this study. Another limitation, similarly noted by Mainz et al. (2024), was the high drop-off rate and the low response rate. Although the response rate was lower in comparison to the average response rates achieved in nursing research (L’Ecuyer et al., 2023), comparable rates have been previously reported (Turunen et al., 2013). Although many nurses accessed the questionnaire online, some did not complete the questionnaire to end. This may have contributed to potential response bias and the over or under-estimation of the frequency of and reasons for missed nursing care. Questionnaire completion fatigue due to frequent research participation invites has been noted in nursing research with many nurses becoming disinterested in participating (White, 2012). Time limitations and high workloads, which are well-known barriers to recruiting nurses (Weierbach et al., 2010), may also have contributed to the small sample size.

The inability to estimate a response rate due to the patient level variation across the study period is a limitation. Another limitation was the lack of a nested sampling strategy to link the patient and nurse responses such as the sampling method used by Cohen et al. (2024). Although the nurses and patients were recruited concurrently from the same wards in the same hospital, allowing the same care period to be captured, the lack of a linked patient and nurse sample limited the ability to make direct comparisons between the nurse and patient perspectives of missed nursing care. Furthermore, using different survey tools to measure the nurse and patient perspectives limited the potential comparison of both viewpoints and contributed to the primarily descriptive aims of this research.

Although nurses were asked to identify the number of nurses, healthcare assistants, patients, admissions and discharges and the number of patients emergencies on their ward, analyses on these data were outside the scope of this study. Further exploration of these data would be of interest given the association between patient-nurse ratios and missed nursing care (Nantsupawat et al., 2022) which demonstrates the potential impact that the number of nurses, healthcare assistants and patients in a ward may have on missed nursing care.

Although the Fundamentals of Care Framework is recognised as being representative of core nursing care, it has some limitations. The framework’s definition of fundamental care has been perceived as being too general and overly idealistic in terms of nurses’ work. Refinement of the framework to include additional clinical elements such as wound care and vital signs is an efficient solution to improve the applicability of the framework to clinical practice (Muntlin et al., 2023). Similarly, this research identified that essential nursing care tasks such as pain management were addressed by the *MISSCARE* survey instruments but not considered directly in the Fundamentals of Care Framework. This reiterates the limitations of the Fundamentals of Care Framework and highlights the potential for its refinement.

Finally, the responses to the questions asked in the *MISSCARE Survey* are based on nurses’ perceptions of missed care rather than their specific experience of carrying out nursing tasks, therefore the responses are influenced by nurses’ beliefs, norms and habits.

A strength of this study was that the participating hospital was a university teaching hospital with a large population of nurses and patients. Furthermore, another strength was the collection of data from various departments and specialties. As using both virtual invites and face-to-face recruitment appeals is a recommended strategy of maximising nurse participation (Raymond et al., 2018), both online and paper-based surveys were used. This was a strength of this study that helped to increase participation by ensuring that the questionnaires were easily accessible and appropriate for all ages and abilities. Although this research is not without limitations, the overall study confirms trends identified by international studies relating to the frequency of and reasons for missed nursing care.

### 4.7 Implications

These findings demonstrate the need to develop a tool to measure missed nursing care from both nurse and patient perspectives (Cohen et al., 2024). This will allow direct comparisons between both perspectives to be drawn and used to identify areas for improving patient safety and healthcare quality. Given the sample size and recruitment challenges, this research highlights the need to consider nurse workload in future measurement tool development. A more sustainable approach to measuring missed nursing care is necessary to elicit adequate response rates. A more succinct and concise measurement tool to consistently measure missed nursing care may be beneficial for identifying areas for improvement and evaluating improvement initiatives. In response to the requirement for a more concise measurement tool, The Situational Nursing Awareness Probe—Missed Nursing Care Edition was developed by Vexler et al. (2025). This tool assesses nurses' situational awareness of missed nursing care, however future research is required to develop a tool to measure and compare both nurse and patient perspectives of missed nursing care.

Finally, this study demonstrates the commonalities between the elements of care in the Fundamentals of Care Framework and the care items investigated through the *MISSCARE* instruments. While the *MISSCARE* instruments can be used to somewhat measure fundamental care delivery, in agreement with Kitson (2018), the development of a specific standardised tool to measure the Fundamentals of Care is warranted.

## 5 Conclusion

This study highlighted the most frequently reported missed nursing care items according to nurses and patients. While nurses are key for delivering high-quality and safe fundamental care, missed nursing care is experienced by patients therefore, including both perspectives of missed nursing care is essential when developing interventions that promote person-centred care. According to patients and nurses, oral care and mobilisation are priority areas for ensuring that fundamental nursing care is delivered. The investigation of the reasons for missed nursing care echoes previous research and reiterates the need to prioritise nurse recruitment and retention strategies. This study also demonstrates the potential of the *MISSCARE* survey instruments to measure elements of the Fundamentals of Care Framework however, further research is warranted to develop a tool to directly measure all three framework dimensions. Furthermore, this study highlights the potential to use the *MISSCARE* surveys to elicit nurses’ and patients’ perspectives of the same phenomena. However, a succinct tool to routinely measure and directly compare both perspectives would be beneficial for developing more targeted interventions to improve patient safety.

## CRediT authorship contribution statement

**Anna Connolly:** Writing – review & editing, Writing – original draft, Methodology, Investigation, Formal analysis, Data curation, Conceptualization. **Anne Matthews:** Writing – review & editing, Supervision, Project administration, Formal analysis, Data curation, Conceptualization. **Marcia Kirwan:** Writing – review & editing, Supervision, Resources, Project administration, Methodology, Funding acquisition, Formal analysis, Data curation, Conceptualization.

## Declaration of competing interest

The authors declare that they have no known competing financial interests or personal relationships that could have appeared to influence the work reported in this paper.

## Appendix Supplementary materials

Image, application 1

Image, application 2

Image, application 3

Image, application 4

Image, application 5

## References

[bib0001] Azzellino G., Dante A., Petrucci C., Caponnetto V., Aitella E., Lancia L., Ginaldi L., De Martinis M. (2025). Intention to leave and missed nursing care: a scoping review. Int. J. Nurs. Stud. Adv..

[bib0002] Bagnasco A., Dasso N., Rossi S., Galanti C., Varone G., Catania G., Zanini M., Aleo G., Watson R., Hayter M., Sasso L. (2020). Unmet nursing care needs on medical and surgical wards: a scoping review of patients’ perspectives. J. Clin. Nurs..

[bib0003] Bail K., Grealish L. (2016). Failure to maintain”: a theoretical proposition for a new quality indicator of nurse care rationing for complex older people in hospital. Int. J. Nurs. Stud..

[bib0004] Boniol M., Kunjumen T., Nair T.S., Siyam A., Campbell J., Diallo K. (2022). The global health workforce stock and distribution in 2020 and 2030: a threat to equity and “universal” health coverage?. BMJ Glob. Health.

[bib0005] Bragadóttir H., Burmeister E.A., Terzioglu F., Kalisch B.J. (2020). The association of missed nursing care and determinants of satisfaction with current position for direct-care nurses—an international study. J. Nurs. Manage.

[bib0006] Cartaxo A., Mayer H., Eberl I., Bergmann J.M. (2024). Missing nurses cause missed care: is that it? Non-trivial configurations of reasons associated with missed care in Austrian hospitals – a qualitative comparative analysis. BMC Nurs..

[bib0007] Chiappinotto S., Coppe A., Palese A. (2023). What are the reasons for unfinished nursing care as perceived by hospitalized patients? Findings from a qualitative study. Health Expect..

[bib0008] Chiappinotto S., Papastavrou E., Efstathiou G., Andreou P., Stemmer R., Ströhm C., Schubert M., de Wolf-Linder S., Longhini J., Palese A. (2022). Antecedents of unfinished nursing care: a systematic review of the literature. BMC Nurs..

[bib0009] Cho S.-H., Kim Y.-S., Yeon K.N., You S.-J., Lee I.D. (2015). Effects of increasing nurse staffing on missed nursing care. Int. Nurs. Rev..

[bib0010] Cho S.-H., Lee J.-Y., You S.J., Song K.J., Hong K.J. (2020). Nurse staffing, nurses prioritization, missed care, quality of nursing care, and nurse outcomes. Int. J. Nurs. Pract..

[bib0011] Cohen M., Drach-Zahavy A., Srulovici E. (2024). Juxtaposing nurses’ and patients’ Perspectives of missed nursing care: a descriptive mixed method design. J. Adv. Nurs..

[bib0012] Constantin S., Dahlke S. (2018). How nurses restore and maintain mobility in hospitalised older people: an integrative literature review. Int. J. Older. People Nurs..

[bib0013] Dabney B.W., Kalisch B.J., Clark M. (2019). A revised MISSCARE survey: results from pilot testing. Appl. Nurs. Res..

[bib0014] Du H., Yang Y., Wang X., Zang Y. (2020). A cross-sectional observational study of missed nursing care in hospitals in China. J. Nurs. Manage.

[bib0015] Feo R., Frensham L.J., Conroy T., Kitson A. (2019). It’s just common sense”: preconceptions and myths regarding fundamental care. Nurse Educ. Pract..

[bib0016] Feo R., Kitson A. (2016). Promoting patient-centred fundamental care in acute healthcare systems. Int. J. Nurs. Stud..

[bib0017] Gallione C., Bassi E., Basso I., Airoldi C., Barisone M., Molon A., Di Nardo G., Torgano C., Dal Molin A. (2024). Missing fundamental nursing care: what’s the extent of missed oral care? A cross-sectional study. Nurs. Rep..

[bib0018] Gil-Montoya J.A., Ferreira de Mello A.L., Barrios R., Gonzalez-Moles M.A., Bravo M. (2015). Oral health in the elderly patient and its impact on general well-being: a nonsystematic review. Clin. Interv. Aging.

[bib0019] Gong F., Mei Y., Wu M., Tang C. (2025). Global reasons for missed nursing care: a systematic review and meta-analysis. Int. Nurs. Rev..

[bib0020] Gurková E., Šinglárová J., Žiaková K. (2024). ‘Nurses’ and patients’ perspectives of missed nursing care in surgical units: a correlation cross-sectional study. Central Eur. J. Nur. Midwif..

[bib0021] Gustafsson N., Leino-Kilpi H., Prga I., Suhonen R., Stolt M. (2020). Missed care from the patient’s perspective – a scoping review. Patient Prefer. Adherence.

[bib65] Kalisch B., McLaughlin M., Marsh V., Nguyen L., Talsma A. (2021). The Development and Testing of the MISSCARE Survey OR. J. Nurs. Measur..

[bib0022] Kalisch B. (2006). Missed nursing care: a qualitative study. J. Nurs. Care Qual..

[bib0023] Kalisch B.J., Doumit M., Lee K.H., Zein J.E. (2013). Missed nursing care, level of staffing, and job satisfaction: Lebanon versus the United States. J. Nurs. Admin..

[bib0024] Kalisch B.J., Landstrom G.L., Hinshaw A.S. (2009). Missed nursing care: a concept analysis. J. Adv. Nurs..

[bib0025] Kalisch B.J., McLaughlin M., Dabney B.W. (2012). Patient perceptions of missed nursing care. Jt. Comm. J. Qual. Patient. Saf..

[bib0026] Kalisch B.J., Tschannen D., Lee H., Friese C.R. (2011). Hospital variation in missed nursing care. Am. J. Med. Qual..

[bib0027] Kalisch B.J., Williams R.A. (2009). Development and psychometric testing of a tool to measure missed nursing care. J. Nurs. Admin..

[bib0028] Kalisch B.J., Xie B., Dabney B.W. (2014). Patient-reported missed nursing care correlated with adverse events. Am. J. Med. Qual..

[bib0029] Kapila Y.L. (2021). Oral health’s inextricable connection to systemic health: special populations bring to bear multimodal relationships and factors connecting periodontal disease to systemic diseases and conditions. Periodontology.

[bib0030] Kitson A., Conroy T., Kuluski K., Locock L., Lyons R. (2013). https://dl.icdst.org/pdfs/files4/3647c83eee8171eb73a269d2a6cb6e1f.pdf.

[bib0031] Kitson A.L. (2018). The Fundamentals of Care framework as a point-of-care nursing theory. Nurs. Res..

[bib0032] Kutschar P., Weichbold M., Osterbrink J. (2019). Effects of age and cognitive function on data quality of standardized surveys in nursing home populations. BMC Geriatr..

[bib0033] L’Ecuyer K.M., Subramaniam D.S., Swope C., Lach H.W. (2023). An integrative review of response rates in nursing research utilizing online surveys. Nurs. Res..

[bib0034] Li F., Goh K.S., Yu X., Png G.K., Chew T.H.S., Ang G.C., Koh X.H., Jose J., Stevenson E. (2025). Mobility matters: a protocol to improve mobility and reduce length of stay in hospitalised older adults. BMJ Open. Qual..

[bib0035] Lim S.H., Ang S.Y., Ong H.K., Lee T.Z.Y., Lee T.X.L., Luo E.Z., Thilarajah S. (2020). Promotion of mobility among hospitalised older adults: an exploratory study on perceptions of patients, carers and nurses. Geriatr. Nurs..

[bib0036] Longhini J., Papastavrou E., Efstathiou G., Andreou P., Stemmer R., Ströhm C., Schubert M., de Wolf-Linder S., Palese A. (2021). Strategies to prevent missed nursing care: an international qualitative study based upon a positive deviance approach. J. Nurs. Manage.

[bib0037] Mainz H., Tei R., Andersen K.V., Lisby M., Gregersen M. (2024). Prevalence of missed nursing care and its association with work experience: a cross-sectional survey. Int. J. Nurs. Stud. Adv..

[bib0038] Merten H., Zegers M., de Bruijne M.C., Wagner C. (2013). Scale, nature, preventability and causes of adverse events in hospitalised older patients. Age Ageing.

[bib0039] Moreno-Monsiváis M.G., Moreno-Rodríguez C., Interial-Guzmán M.G. (2015). Missed nursing care in hospitalized patients. Aquichan.

[bib0040] Morris R., Matthews A., Scott A., Rafferty A.M., Busse R., Zander-Jentsch B., Sermeus W., Bruyneel L. (2019). Strengthening Health Systems Through Nursing: Evidence from 14 European Countries.

[bib0041] Muntlin Å., Jangland E., Laugesen B., Voldbjerg S.L., Gunningberg L., Greenway K., Merriman C., Grønkjær M., Heinen M., Huisman-de Waal G. (2023). Bedside nurses’ perspective on the Fundamentals of Care framework and its application in clinical practice: a multi-site focus group interview study. Int. J. Nurs. Stud..

[bib0042] Nahasaram S.T., Ramoo V., Lee W.L. (2021). Missed nursing care in the Malaysian context: a cross-sectional study from nurses’ perspective. J. Nurs. Manage..

[bib0043] Nantsupawat A., Poghosyan L., Wichaikhum O.-A., Kunaviktikul W., Fang Y., Kueakomoldej S., Thienthong H., Turale S. (2022). Nurse staffing, missed care, quality of care and adverse events: a cross sectional study. J. Nurs. Manage..

[bib0044] Palese A., Longhini J., Danielis M. (2021). To what extent Unfinished Nursing Care tools coincide with the discrete elements of the Fundamentals of Care Framework? A comparative analysis based on a systematic review. J. Clin. Nurs..

[bib0045] Pene B.-J., Aspinall C., Komene E., Slark J., Gott M., Robinson J., Parr J.M. (2025). The Fundamentals of care in practice: a qualitative contextual inquiry. Nurs. Inq..

[bib0046] Phillips J., Peck Malliaris A., Bakerjian D. (2021). Nursing and patient safety. PSNet.

[bib0047] Raymond C., Profetto-McGrath J., Myrick F., Strean W.B. (2018). Process matters: successes and challenges of recruiting and retaining participants for nursing education research. Nurse Educ..

[bib0048] Rezaei-Shahsavarloo Z., Atashzadeh-Shoorideh F., Ebadi A., Gobbens R.J.J. (2021). Factors affecting missed nursing care in hospitalized frail older adults in the medical wards: a qualitative study. BMC Geriatr..

[bib0049] Rezaei-Shahsavarloo Z., Atashzadeh-Shoorideh F., Gobbens R.J.J., Ebadi A., Ghaedamini Harouni G. (2020). The impact of interventions on management of frailty in hospitalized frail older adults: a systematic review and meta-analysis. BMC Geriatr..

[bib0050] Sarpong A.A., Towell-Barnard A., Gent L., Afrifa-Yamoah E., Arabiat D. (2025). Patients’ Perception of missed nursing care in a tertiary hospital: a cross-sectional study. Nurs. Open.

[bib0051] Senek M., Robertson S., Ryan T., King R., Wood E., Tod A. (2020). The association between care left undone and temporary nursing staff ratios in acute settings: a cross- sectional survey of registered nurses. BMC Health Serv. Res..

[bib0052] Serdar C.C., Cihan M., Yücel D., Serdar M.A. (2021). Sample size, power and effect size revisited: simplified and practical approaches in pre-clinical, clinical and laboratory studies. Biochem. Med..

[bib0053] Sheehan A., McGarrigle C., O’Connell B. (2017).

[bib0054] Turunen H., Partanen P., Kvist T., Miettinen M., Vehviläinen-Julkunen K. (2013). Patient safety culture in acute care: a web-based survey of nurse managers’ and registered nurses’ views in four Finnish hospitals. Int. J. Nurs. Pract..

[bib0055] Vexler M., Drach-Zahavy A., Srulovici E. (2025). Development of a questionnaire assessing nurses’ Situational awareness to missed care. Nurs. Health Sci..

[bib0056] Weierbach F.M., Glick D.F., Fletcher K., Rowlands A., Lyder C.H. (2010). Nursing research and participant recruitment: organizational challenges and strategies. J. Nurs. Adm..

[bib0057] White E. (2012). Challenges that may arise when conducting real-life nursing research. Nurse Res..

[bib0058] World Health Organization (2020). https://iris.who.int/handle/10665/331677.

[bib0059] World Health Organization (2024). https://www.who.int/health-topics/nursing.

[bib0060] World Health Organization (2024). https://www.who.int/news-room/fact-sheets/detail/nursing-and-midwifery.

[bib0061] World Medical Association (2018). https://www.wma.net/policies-post/wma-declaration-of-helsinki/.

[bib0062] Zelenikova R., Gurkova E., Jarosova D. (2019). Missed nursing care measured by MISSCARE Survey – the first pilot study in the Czech Republic and Slovakia. Centr. Eur. J. Nurs. Midwif..

[bib0063] Zhu X., Zheng J., Liu K., You L. (2019). Rationing of nursing care and its relationship with nurse staffing and patient outcomes: the mediation effect tested by structural equation modeling. Int. J. Environ. Res. Public Health.

