# Supplementary material for: Nurses’ and older patients’ perspectives on missed nursing care contextualised within the Fundamentals of Care Framework: A cross-sectional survey
Source: Int J Nurs Stud Adv. 2025 Nov 11;9:100452. doi: 10.1016/j.ijnsa.2025.100452 (PMC12666513; doi:10.1016/j.ijnsa.2025.100452)
Supplement: Supplementary file 1 [file mmc1.docx]

**Supplementary figure 1.**

Figure 1: Reasons for missed care according to nurses
