# Supplementary material for: Nurses’ and older patients’ perspectives on missed nursing care contextualised within the Fundamentals of Care Framework: A cross-sectional survey
Source: Int J Nurs Stud Adv. 2025 Nov 11;9:100452. doi: 10.1016/j.ijnsa.2025.100452 (PMC12666513; doi:10.1016/j.ijnsa.2025.100452)
Supplement: Supplementary file 2 [file mmc2.docx]

**Nurse Survey**

ABOUT YOU

1. What is your gender?

Female Male Other Prefer not to say

1. What is your age? Years: ______
2. What is your job title/role?

Staff Nurse (RN) ☐

Nurse Manager (e.g., Clinical Nurse Manager)

Other [Please specify: ____________]

1. A) Did you receive your basic nursing education in Ireland? _________

B) If no, in what country did you receive your basic nursing education? Country ­­­: _________

1. Do you have a baccalaureate (level 8) degree in nursing?

Yes No

1. How satisfied are you with your choice of nursing as a career?

Very dissatisfied A little dissatisfied Moderately satisfied Very satisfied

1. How many years have you worked as a registered nurse…
2. in your career Years:______ Months: _______
3. in this hospital Years:______ Months: _______
4. For how many years have you worked as a registered nurse…
5. in the specialty in which you currently work Years:______ Months: _____
6. Did in this ward/unit? Years:______ Months: _____

ABOUT YOUR MOST RECENT SHIFT AT WORK IN THIS HOSPITAL

1. Please indicate the type of unit you work on:

Surgical (any surgical specialty) ☐

Medical (any medical specialty) ☐

Mixed Medical/Surgical ☐

Intensive Care Unit (ICU) ☐

High Dependency Unit (HDU) ☐

Other [Please specify: ____________

1. On your most recent shift how many patients in total were on your unit/ward? ________
2. Counting yourself, how many registered nurses in total provided direct patient care on your unit/ward during the most recent shift you worked?

Number of registered nurses: ­­­________

1. How many other nursing care staff (Healthcare assistants) in total provided direct patient care on your unit/ward during the most recent shift you worked?

Other nursing care staff: _______

1. A) How many patient-admissions did you have on your most recent shift (i.e., includes transfers into the unit/ward)? __________
2. B) How many patient-discharges did you have on your most recent shift (i.e., includes transfers out of the unit/ward)? ________
3. C) How many patient emergencies did you have on your most recent shift (i.e., falls, deteriorating clinical condition of patient, etc.) ________

**Section A**

Nursing staff frequently encounter multiple demands on their time, requiring them to reset priorities. To the best of your knowledge, how frequently are the following nursing care tasks left undone by nursing staff at the end of a shift due to these multiple demand on your unit/ward? Check only one box for each item.

|  | **Always**  **Missed** | **Frequently Missed** | **Occasionally Missed** | **Rarely Missed** | **Never Missed** | **Not Applicable** |
| --- | --- | --- | --- | --- | --- | --- |
| 1) Mobilization |  |  |  |  |  |  |
| 2) Pressure relieving interventions |  |  |  |  |  |  |
| 3) Feeding patients while food is still at the proper temperature |  |  |  |  |  |  |
| 4) Setting up meals for patients who can feed themselves |  |  |  |  |  |  |
| 5) Medications administered as scheduled and on time |  |  |  |  |  |  |
| 6) Assessment of vital signs |  |  |  |  |  |  |
| 7) Monitoring intake/output |  |  |  |  |  |  |
| 8) Full documentation of all care provided |  |  |  |  |  |  |
| 9) Bathing/Showering |  |  |  |  |  |  |
| 10) Oral care |  |  |  |  |  |  |
| 11) Glucose monitoring as ordered |  |  |  |  |  |  |
| 12) IV/central line site care and assessments according to hospital policy |  |  |  |  |  |  |
| 13) Response to call light is initiated within 5 minutes |  |  |  |  |  |  |
| 14) PRN medication requests acted on within 15 minutes |  |  |  |  |  |  |
| 15) Attend interdisciplinary care conferences when held |  |  |  |  |  |  |
| 16) Assist with toileting needs within 5 minutes of request |  |  |  |  |  |  |
| 17) Skin/Wound care |  |  |  |  |  |  |
| 18) Adequate surveillance of cognitively impaired patients |  |  |  |  |  |  |

**Section B—Reasons**

Thinking about the care left undone on your unit/ward/shift by nursing staff (as you indicated the section above), please assess the contribution of each of the statements below. Check only one box for each item.

|  | **Significant**  **Contribution** | **Moderate**  **Contribution** | **Minor**  **Contribution** | **No Contribution** |
| --- | --- | --- | --- | --- |
| 1) Inadequate number of staff |  |  |  |  |
| 2) Urgent patient situations (e.g., a patient’s condition worsening, patient fall) |  |  |  |  |
| 3) Unexpected rise in acuity on the unit |  |  |  |  |
| 4) Inadequate number of assistive personnel (e.g., healthcare assistants, etc.) |  |  |  |  |
| 5) Unbalanced patient assignments |  |  |  |  |
| 6) Medications not available when needed |  |  |  |  |
| 7) Inadequate hand-over from previous shift or transferring ward/unit |  |  |  |  |
| 8) Other departments did not provide the care needed (e.g., physical therapy (physiotherapy), occupational therapy, medical social work) |  |  |  |  |
| 9) Supplies/ equipment not available when needed |  |  |  |  |
| 10) Supplies/ equipment not functioning properly when needed |  |  |  |  |
| 11) Lack of back up support from team members |  |  |  |  |
| 12) Tension or communication breakdowns with other staff/departments |  |  |  |  |
| 13) Tension or communication breakdowns within the nursing team or with the medical staff |  |  |  |  |
| 14) Inadequate support from nursing leadership |  |  |  |  |
| 15) Heavy admission and discharge activity |  |  |  |  |
| 16) Emotional or physical exhaustion |  |  |  |  |
| 17) Inadequate supervision of healthcare assistants |  |  |  |  |
| 18) Interruptions/Multitasking |  |  |  |  |
| 19) Lack of cues/reminders |  |  |  |  |

QUALITY AND SAFETY

1. In general, how would you describe the quality of nursing care delivered to patients on your unit/ward?

Poor  Fair  Good  Excellent

1. Please give your unit/ward an over grade on patient safety.

Poor  Fair  Good  Excellent

1. How often do you feel the unit staffing is adequate?

100% of the time ☐

75% of the time ☐

50% of the time ☐

25% of the time ☐

0% of the time ☐

**THANK YOU FOR YOUR PARTICIPATION!**

**Patient Survey**

To the extent you can remember, please answer the following questions, if you cannot remember, leave the answer blank.

1. Please select one option in response to each question.

|  | **Never** | **Rarely** | **Sometimes** | **Usually** | **Always** |
| --- | --- | --- | --- | --- | --- |
| How often were you clear about which specific nurse was assigned to take care of you for the shift? |  |  |  |  |  |
| How often did your nursing staff discuss your treatment with you? |  |  |  |  |  |
| How often did your nursing staff give you information about tests (e.g. x-ray, MRI, CT scan) and/or procedures you received during this hospitalization (timing, what would be involved, etc.)? |  |  |  |  |  |
| When you had a question or concern about your care or illness, did your nursing staff listen to you? |  |  |  |  |  |
| When you had an opinion about what needed to be done relative to your care, did the nursing staff consider your opinions and ideas? |  |  |  |  |  |
| How often did the nursing staff check with you to make sure your teeth were brushed/cleaned and mouth rinsed (or provide the care if you could not do it yourself)? |  |  |  |  |  |
| How often did the nursing staff check with you to make sure you had a shower/wash throughout your hospitalization? |  |  |  |  |  |

1. If you could not feed yourself at any time during your hospitalization, did the nursing staff help to feed you within 10 minutes after the arrival of the tray?

Never

Rarely

Sometimes

Usually

Always

Check here if you did not need help to feed yourself

Check here if you could not eat

1. On average, how often did the nursing staff help you or monitor that you got out of bed and sat in a chair?

Never

Rarely

Sometimes

Usually

Always

Check here if you were unable to get out of bed

1. On average, how often did the nursing staff help you or monitor that you walked?

Never

Rarely

Sometimes

Usually

Always

Check here if you could not walk

1. On average, how often did the nursing staff reposition you in bed?

Never

Rarely

Sometimes

Usually

Always

Check here if you did not need help moving around in bed

1. On average, how often did your nurses check your IV or other line (central venous catheter, PICC line, or port)?

Never

Rarely

Sometimes

Usually

Always

Check here if you did not have an iv or other line

1. When a monitor or other machine beeped, how long did it usually take the nursing staff to respond?

Less than 5 minutes

5 to 10 minutes

11 to 20 minutes

21 to 30 minutes

More than 30 minutes

No machine beeped

1. When you pressed your call light, how long on average did it take the nursing staff to answer?

Less than 5 minutes

5 to 10 minutes

11 to 20 minutes

21 to 30 minutes

More than 30 minutes

I never pressed my call light

1. Once your call light was answered, how long on average did it take for you to receive the help you requested?

Less than 5 minutes

5 to 10 minutes

11 to 20 minutes

21 to 30 minutes

More than 30 minutes

I never pressed my call light

1. Did you ask for pain medication?

YES (if yes, go to question 11)

NO (if no, go to question 13)

1. If you answered yes to question 10, how long did it take you to get the pain medication?

Less than 5 minutes

5 to 10 minutes

11 to 20 minutes

21 to 30 minutes

More than 30 minutes

I never received the pain medication

1. If you answered yes to question 10, did the nursing staff check back to see if the medication helped reduce your pain?

Never

Rarely

Sometimes

Usually

Always

1. If you needed help to go to the bathroom, how long did it take the nursing staff to get into your room to help you?

Less than 5 minutes

5 to 10 minutes

11 to 20 minutes

21 to 30 minutes

More than 30 minutes

I did not request or need help

1. Overall, how would you rate your nursing care while you were a patient during this hospitalization?

Poor

Fair

Good

Very good

Excellent

1. Please select one option in response to each question.

|  | **Yes** | **No** | **Unsure** |
| --- | --- | --- | --- |
| Did you experience a fall during this hospitalisation? |  |  |  |
| Did you experience a pressure ulcer during this hospitalisation? |  |  |  |
| Was your medication always correct? |  |  |  |
| Was your medication always on time? |  |  |  |
| Did you get any infection during this hospitalisation? |  |  |  |
| Did you experience any problems with your IV cannula during this hospitalisation? |  |  |  |
| Did your cannula have to be replaced during this hospitalisation? |  |  |  |

**General Information**

1. Type of ward

Surgical (any surgical specialty) ☐

Medical (any medical specialty) ☐

Mixed Medical/Surgical ☐

Intensive Care Unit (ICU) ☐

High Dependency Unit (HDU) ☐

Other [Please specify: ____________

1. How many days have you been in the hospital this time? ____ days Unsure
2. Have you been hospitalized before? Yes No
3. How many times in the last 5 years?_____ Unsure
4. Age: ________ years old
5. Gender:  Male  Female  Other  Prefer not to say
6. In general, how would you rate your overall health?

Poor  Fair  Good  Very good  Excellent

**THANK YOU FOR YOUR PARTICIPATION!**
