# Supplementary material for: Nurses’ and older patients’ perspectives on missed nursing care contextualised within the Fundamentals of Care Framework: A cross-sectional survey
Source: Int J Nurs Stud Adv. 2025 Nov 11;9:100452. doi: 10.1016/j.ijnsa.2025.100452 (PMC12666513; doi:10.1016/j.ijnsa.2025.100452)
Supplement: Supplementary file 3 [file mmc3.docx]

Supplementary Table 1: Nurse characteristics

| Nurse Characteristics |  |
| --- | --- |
| Gender, *n* (%) |  |
| Male | 21 (14.0%) |
| Female | 129 (86.0%) |
| Age, mean (SD) | 37.69 (9.840) |
| Job title/role, *n* (%) |  |
| Staff nurse (RN) | 117 (78.0%) |
| Nurse Manager/Senior role | 33 (22.0%) |
| Received nursing education in Ireland, *n (%)* |  |
| Yes | 45 (30.0%) |
| No | 105 (70.0%) |
| Baccalaureate (level 8) degree in nursing, *n (%)* |  |
| Yes | 130 (87.8%) |
| No | 18 (12.2%) |
| Unit Worked on, *n (%)* |  |
| Surgical | 16 (10.6%) |
| Medical | 64 (42.4%) |
| Mixed Medical/Surgical | 12 (7.9%) |
| A&E | 2 (1.3%) |
| Critical Care | 27 (17.9%) |
| Other | 30 (19.9%) |
| Experience, *mean (SD)* |  |
| Years worked as a registered nurse in career | 13.64 (9.84) |
| Job satisfaction, *n (%)* |  |
| Very dissatisfied | 13 (8.8%) |
| A little dissatisfied | 13 (8.8%) |
| Moderately satisfied | 41 (27.9%) |
| Very satisfied | 80 (54.4%) |
