# Supplementary material for: Nurses’ and older patients’ perspectives on missed nursing care contextualised within the Fundamentals of Care Framework: A cross-sectional survey
Source: Int J Nurs Stud Adv. 2025 Nov 11;9:100452. doi: 10.1016/j.ijnsa.2025.100452 (PMC12666513; doi:10.1016/j.ijnsa.2025.100452)
Supplement: Supplementary file 4 [file mmc4.docx]

Supplementary Table 2: Patient characteristics

| Patient Characteristics |  |
| --- | --- |
| Gender, *n (%)* |  |
| Male | 96 (68.1%) |
| Female | 45 (31.9%) |
| Age, mean (SD) | 75.11 (6.822) |
| Unit, *n (%)* |  |
| Surgical | 30 (22.60%) |
| Medical | 66 (49.6%) |
| Mixed Medical/Surgical | 14 (10.5%) |
| Critical Care | 1 (1.5%) |
| Other | 21 (15.8%) |
| Days in hospital, mean (range) | 23.99 (3-300) |
| Previous hospitalisation, *n (%)* |  |
| Yes | 129 (92.1%) |
| No | 11 (7.9%) |
| Self-reported rating of overall health, *n (%)* |  |
| Poor | 15 (10.8%) |
| Fair | 38 (27.3%) |
| Good | 58 (41.7%) |
| Very Good | 18 (12.9%) |
| Excellent | 10 (7.2%) |
|  |  |
