# Supplementary material for: Nurses’ and older patients’ perspectives on missed nursing care contextualised within the Fundamentals of Care Framework: A cross-sectional survey
Source: Int J Nurs Stud Adv. 2025 Nov 11;9:100452. doi: 10.1016/j.ijnsa.2025.100452 (PMC12666513; doi:10.1016/j.ijnsa.2025.100452)
Supplement: Supplementary file 5 [file mmc5.docx]

Supplementary Table 3: The number of nurses and healthcare assistants who provided care, the number of patients cared for, the number of admissions and discharges and the number of patients emergencies on the wards within which the nurse participants work

|  | n | Minimum | Maximum | Mean | SD |
| --- | --- | --- | --- | --- | --- |
| On your most recent shift how many patients in total were on your ward/unit? | 149 | 4 | 35 | 21.19 | 9.097 |
| Counting yourself, how many registered nurses in total provided direct patient care on your unit/ward during the most recent shift you worked? | 123 | 1 | 8 | 4.48 | 1.479 |
| How many other nursing care staff (Healthcare assistants) in total provided direct patient care on your unit/ ward during the most recent shift you worked? | 131 | 0 | 3 | 1.64 | 0.724 |
| How many patient-admissions did you have on your most recent shift (i.e., includes transfers into the unit/ward)? | 143 | 0 | 8 | 2.45 | 1.814 |
| How many patient-discharges did you have on your most recent shift (i.e., includes transfers out of the unit/ward)? | 126 | 0 | 4 | 1.65 | 1.195 |
| How many patient emergencies did you have on your most recent shift (i.e., falls, deteriorating clinical condition of the patient, etc.) | 142 | 0 | 4 | 1.10 | 1.061 |
